# Online extremism and the communities that sustain it: Detecting the ISIS supporting community on Twitter

**DOI:** 10.1371/journal.pone.0181405

**Published:** 2017-12-01

**Authors:** Matthew C. Benigni, Kenneth Joseph, Kathleen M. Carley

**Affiliations:** 1 Institute for Software Research, School of Computer Science, Carnegie Mellon University, Pittsburgh, PA, United States of America; 2 Network Science Institute, Northeastern University, Boston, MA, United States of America; 3 Institute for Quantitative Social Science, Harvard University, Cambridge, MA, United States of America; Universitat Rovira i Virgili, SPAIN

## Abstract

The Islamic State of Iraq and ash-Sham (ISIS) continues to use social media as an essential element of its campaign to motivate support. On Twitter, ISIS’ unique ability to leverage unaffiliated sympathizers that simply retweet propaganda has been identified as a primary mechanism in their success in motivating both recruitment and “lone wolf” attacks. The present work explores a large community of Twitter users whose activity supports ISIS propaganda diffusion in varying degrees. Within this ISIS supporting community, we observe a diverse range of actor types, including fighters, propagandists, recruiters, religious scholars, and unaffiliated sympathizers. The interaction between these users offers unique insight into the people and narratives critical to ISIS’ sustainment. In their entirety, we refer to this diverse set of users as an online extremist community or OEC. We present Iterative Vertex Clustering and Classification (IVCC), a scalable analytic approach for OEC detection in annotated heterogeneous networks, and provide an illustrative case study of an online community of over 22,000 Twitter users whose online behavior directly advocates support for ISIS or contibutes to the group’s propaganda dissemination through retweets.

## Introduction

Through an effective social media campaign, the Islamic State of Iraq and ash-Sham (ISIS) has issued a powerful, global call to arms. On Youtube, Twitter and a host of other social media platforms, an ethnically diverse set of Jihadists issue a similar calls to would be fighters living in the West. Their message promises heaven to those who answer their call to arms. This strategy initially was used to motivate foreign fighters to join ISIS campaigns in Syria and Northern Iraq [1], but online radicalization appears to be a critical component of the groups shift toward decentralized attacks in the West [2].

Online Extremism can be defined as advocating support of groups or causes that in any distribution of opinion would lie on one of the “tails” [3]. Although the methods and ends espoused by ISIS’ online marketing campaign clearly meet the definition of extremism, the campaign’s global reach has generated an operationally significant amount of online and offline support. As of January, 2015, United States intelligence sources estimate ISIS had between 9,000 and 18,000 fighters in Iraq and Syria [4]. Although the majority of ISIS’ fighters are from the Middle East and North Africa (MENA), a surprising number of fighters have arrived from the Western world. ISIS’ message has global reach and has even motivated lone wolf attacks in Canada [5], France [6], the United States [7], and the United Kingdom [8].

Not all members of ISIS’ online community display the same levels of online extremism. Some claim unaffiliated sympathizers who simply retweet or repost propaganda represent a paradigmatic shift explaining ISIS’ unprecedented online success [9–12]. In many cases these unaffiliated users’ activity, although offensive to many, is not in clear violation of law or “The Twitter Rules [13].” However, this large body of “passive supporters” contribute to the volume of ISIS related content proliferated on Twitter and appears to be a vital component of ISIS social media campaign. These individuals are therefore of interest to any effort to counter online extremism. Some of these passive sympathizers become recruiting targets. ISIS uses small teams of social media users to lavish attention on the potential recruits and move the conversation to more secure online platforms [12]. Thus, while Twitter may not be the place where recruitment ends, growing evidence suggests that identifiable patterns of recruitment *begin* on Twitter.

The primary goal of this work is to provide methods allowing researchers to gain insight into this online social network of unaffiliated sympathizers, propagandists, fighters and recruiters, and how these users interact to create a thriving *online extremist community* (OEC). We argue that such understanding is needed to create counter-narratives tailored to the online populations most vulnerable to this type of online extremism. To do so, we must first solve another problem—identifying an OEC on Twitter. This task is difficult for three reasons. First, the size of OECs varies and is often unknown. With respect to ISIS, it has been estimated that the OEC is between 46,000 and 70,000 strong [11]. However, the relatively small intersection between existing datasets maintained by activists and researchers indicates the group could in fact be much larger. Second, current social media community detection methods require a great deal of manual intervention, or provide unacceptable precision via automated methods—there is thus an existing tradeoff between manual coding of the data and highly inaccurate classification tools in the existing literature.

As ISIS’ popularity has grown, so too has its opposition; thus the ISIS OEC and extremist groups in general tend to be *covert* in that they actively attempt to avoid some form of detection. Twitter now systematically identifies and suspends user accounts associated with the group [14]. In fact, Twitter has initiated a systematic campaign to neutralize ISIS’ use of the site and announced in March of 2016 the suspension of over 125,000 ISIS supporting accounts in a six month period [15]. Furthermore, activist groups like Anonymous and Lucky Troll Club have used crowd sourcing to identify and expose ISIS OEC members on Twitter [16–18]. These attempts to limit ISIS’ use of social media platforms has resulted in a predator-prey-like system where the ISIS OEC on Twitter has begun show systematic attempts to make the network anonymous and resilient.

Our work makes three major contributions to the literature. First, we present Iterative Vertex Clustering and Classification (IVCC), a novel approach to detect and extract knowledge from OECs. Our approach utilizes community optimization methods in conjunction with *multiplex vertex classification (MVC)*, a classification method used on heterogeneous graphs that leverages the rich data structures common to many OSNs like user meta-data, mentioning, following, and hash tag use. Capitalizing on this rich structure enables us to outperform existing methods with respect to recall and precision which will be shown in Section 4.

After considering the merits of our approach, we then turn to the second major contribution of this work, an illustrative case study of the ISIS OEC on Twitter. By searching known members’ following ties and partitioning the resultant network, we identify a community of over 22,000 Twitter users whose online behavior contributes to the online proliferation of ISIS propaganda. We leverage clustering and Twitter suspensions to infer positive case instances with our classifier which is able to partition our training set with 96% accuracy. This offers significant improvement over existing methods, and we claim this makes our output uniquely valid for the study of online radicalization.

Finally, we discuss an ethical framework for the implementation of methods similar to IVCC. We highlight the framework presented in [19] of: methods, context, and target, and we draw distinctions in context between diplomatic and intelligence applications of social media mining.

We structure this article as follows: In Section 1 we discuss related work and highlight the limitations of common community detection methodologies with respect to OEC detection. Section 2 provides a detailed overview of our proposed community detection methodology, followed by an illustrative case study of the ISIS OEC on Twitter in Section 3. Section 4 provides a detailed discussion of the relative performance of IVCC, and Section 5 provides a case study of the ISIS supporting OEC on Twitter and illustrative knowledge extractions useful for counter-messaging or intelligence purposes. We then discuss the societal implications and limitations associated with the potential uses of our methods in Section 6, and propose future research in Section 7.

## 1 Background

Krebs [20, 21] was the first to cast large-scale attention on network science-based counter-terrorism analysis with his application of network science techniques to gain insight into the September 11, 2001 World Trade Center Bombings. Although similar methods were presented years earlier [22], the timeliness of Krebs’ work caught the attention of the Western world and motivated a great deal of further research[23–29]. Much of this work focused on constructing networks based on intelligence and using the network’s topology to identify key individuals and evaluate intervention strategies. The rise of social media has introduced new opportunities for network science-based counter-terrorism, and some foresee social media intelligence *(SOCMINT)* as being a major intelligence source in the future [30]. This presents a fundamentally different counter-terrorism network science problem. Roughly, as opposed to using information about individuals to build networks, we now use networks to gain insight into individuals. Typically, we are also trying to identify a relatively small and possibly covert community within a much larger network. Such a change requires methodologies optimized to detect covert networks embedded in social media.

The problem of community detection has been widely studied within the context of large-scale social networks [31]. Community detection algorithms attempt to identify groups of vertices more densely connected to one another than to the rest of the network. Social networks extracted from social media, however, present unique challenges due to their size and high clustering coefficients [32]. Furthermore, ties in online social networks like Twitter are widely recognized to represent different types of relationships [33–36].

The algorithms of Newman [37] and Blondel [38] are recognized as a standard for comparison for community detection within network science. Within the broad landscape of all community detection algorithms, the work of both Newman and Blondel fall under the umbrella of what is more accurately referred to as community optimization algorithms. In community optimization algorithms, the graph is partitioned into *k* communities based on an optimization problem that centers around minimizing inter-community connections are minimized and *k* is unspecified. Surprisingly, both Newman and Blondel operationalize this minimization problem as a maximization one, where they maximize *modularity*. The modularity of a graph is defined in Eq 1. In Eq 1, the variable *A*_*i*,*j*_ represents the weight of the edge between nodes *i* and *j*, *k*_*i*_ = ∑_*j*_ *A*_*i*,*j*_ is the sum of the weights of the edges attached to vertex *i*, *c*_*i*_ is the community to which vertex *i* is assigned, *δ*(*u*, *v*) is the inverse identity function, and $m=\frac{1}{2}\sum_{i,j}A_{i,j}$.

$$\begin{array}{c} Q=\frac{1}{2m}\sum_{i,j}=\left[A_{i,j}-\frac{k_{i}k_{j}}{2m}\right]\delta \left(c_{i},c_{j}\right), \end{array}$$ (1)

Eaton and Mansbach [39] have introduced methods from constrained clustering literature to enable semi-supervised community optimization where a subset of vertices have known memberships as well. While such algorithms work well for certain classes of problems, community optimization algorithms have shown limited ability to detect threat networks embedded in social media when the group maintains connections with non-group members [35]. Community optimization is also unable to effectively account for multiplex graphs or graphs with multiple connection types. Like community optimization, graph partitioning finds partitions by minimizing intra-group connections; however, the number of groups, *k*, is fixed [31]. Covert network detection is then best described as a special case of graph partitioning where the partition is binary (or in other words, where *k* = 1) [40]. Smith et al. [40] effectively use this viewpoint to model spatiotemporal threat propagation using Bayesian inference, however their method does not extent to multiplex or multimode graphs when applied to social media. To do so, other methods must be used.

In recent years, another sub-class of community detection methods has emerged, community detection in annotated networks. This body of work attempts to effectively incorporate node level attributes into clustering algorithms to account for noisiness of social networks embedded in social media. Vertex clustering originates from traditional data clustering methods and embeds graph vertices in a vector space where pairwise, Euclidian distances can be calculated [31]. In such approaches, a variety of eigenspace graph representations are used with conventional data clustering and classification techniques such as K-means or hierarchical agglomerative clustering, and support vector machines. These methods offer the practitioner great flexibility with respect to the types of information used as features. Vertex clustering and classification methods have been shown to perform well with social media because of their ability to account for a great variety of vertex features like user account attributes while still capitalizing on the information embedded in the graph; they also perform well at scale [34, 41]. [34] introduces a vertex clustering framework, *SocioDim*, which detects communities embedded in social media by performing vertex clustering where network features are represented spectrally and paired with user account features. Very similar methods are also presented in [42]. [41] then applies *SocioDim* to classification, which is analogous to a binary partition of the graph.

These methods show clear promise with respect to covert network detection in social media as illustrated by [35]. Eigenspace methods have been shown to adequately model multiplex representations of various types of social ties in social media [43], and early studies of simulated networks indicate they would perform well on threat detection in social media [35]. We hypothesize that eigenspace representations of multiplex social networks embedded in social media, when paired with user account features and node level features will provide a more powerful means to detect extremist communities embedded in social media. Our work utilizes community optimization across multiple graphs in an annotated heterogeneous network to facilitate vertex classification and detect a targeted covert community. In sum, we have found that each of the methods listed above offer useful information for classification, but a combination of these techniques must be used to effectively detect covert networks embedded in social media.

## 2 Methods: Iterative Vertex Clustering and Classification

The goal of finding an OEC within a larger dataset can be formalized as attempting to find a relatively small subgraph within a large, annotated, heterogeneous network, *G* = (*V*_1_, *V*_2_, .., *V*_*n*_, *E*_1_, *E*_2_, .., *E*_*m*_). The full network *G* is a directed, weighted graph with vertex sets *V*_1_…*V*_*n*_. Each vertex set contains vertices *v*_*n*,1_..*v*_*n*,*j*_ with one or more edge types *E*_1_, *E*_2_, .., *E*_*m*_. We define a subset of targeted vertices *A*_*t*_ ⊆ *V*_*t*_ and denote its complement as $\tilde{A_{t}}$. Our goal is to accurately classify each vertex in *V*_*t*_ as members of either *A*_*t*_ or $\tilde{A_{t}}.$ For example, in our case study we define *A*_*t*_ as our set of *ISIS OEC members* and $\tilde{A_{t}}$ as the union of both *non-members* and *Official Accounts*, which will be defined below.

**Fig 1:**
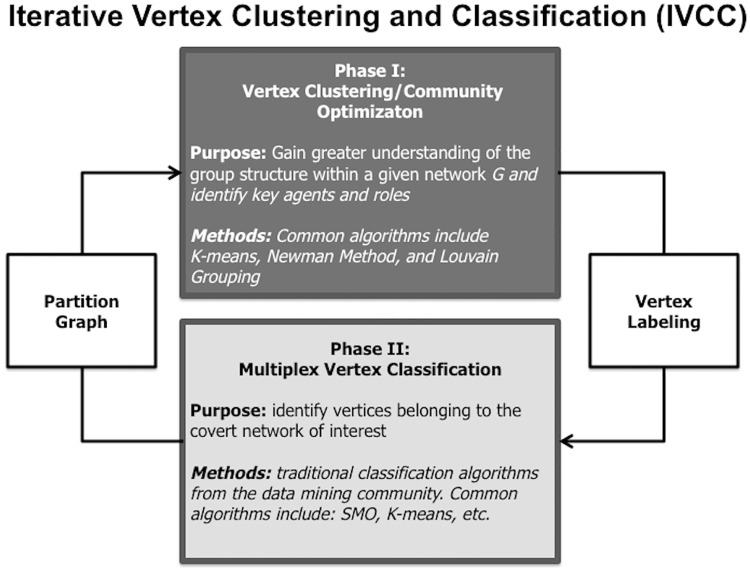
We present an iterative methodology conducted in two phases. In Phase I either community optimization or vertex clustering algorithms are used to remove noise and facilitate supervised machine learning to partition vertices in Phase II.

In practice, we will often have partial knowledge of the group and its members, and need to leverage as much information as possible to identify vertices in *A*_*t*_. Our methodology leverages a priori knowledge to search for and detect a covert subgraph in social media by iteratively utilizing community optimization and vertex classification. Our approach is thus conducted in two phases. In Phase I, community optimization algorithms and a priori knowledge are used to gain insight into the larger social network and facilitate supervised machine learning in Phase II. Phase II partitions vertices, retaining only those in *A*_*t*_, thus finding the targeted covert community. A diagram of the process can be seen in Fig 1.

### 2.1 Phase I: Vertex clustering and community optimization

Although community optimization and vertex clustering methods will often fail to accurately partition our networks into *A*_*t*_ and $\tilde{A_{t}}$[35], we can often look for community structure within the network to gain insight into the set of vertices in *A*_*t*_. For example, if a subset of vertices from *A*_*t*_ is known, community optimization can identify clusters containing a large proportion of those known vertices belonging to *A*_*t*_. Community optimization can also identify groups of vertices that are clearly members of $\tilde{A_{t}}$. The insights gained from community optimization help provide necessary context with respect to algorithm selection and case labels for vertex classification in Phase II of our methodology.

### 2.2 Phase II: Multiplex vertex classification

Like [41] we classify *v*_*t*,1_…*v*_*t*,*j*_ using a set of features extracted from the users’ social media profiles and spectral representations of the multiplex ties between *V*_*t*_. We denote these spectral representations as *U*_*V*_*t*_×*V*_*t*_;*E*_*i*__, where *i* = 1, …, *m*. To develop spectral representations of our heterogeneous network, we symmetrize the graphs *W* = *G*_*V*_*n*_×*V*_*n*_;*E*_*m*__ for ∀*E*_*m*_. These symmetric graphs also leverage the strength of reciprocal ties, which have been shown to better indicate connection in social networks embedded in social media [44–46]. In our case study we refer to the symmetrized network of following ties as *F*_*rec*_, and the symmetrized network of mention ties as *M*_*rec*_. We then extract the eigenvectors of the graph Laplacian associated with the smallest two eigenvalues as highlighted in [47], and we concatenate these matrices as presented in [43]. This enables us to effectively capture the distinct ties represented in many types of social media, as well as node level metrics of each graph and user account features.

Users can often use topical markers like hash tags in Twitter, and these can be used to cluster users with similar topical interests. This results in bipartite graphs, *G*_*V*_*t*_×*V*_*n*_,*E*_*m*__, where users and topical markers represent differing node sets, however we with to use these links to find similarities with respect to topical interests among users. To do so we implement bispectral clustering as introduced by [48] as a document clustering method. In our case, instead of co-clustering documents based on word frequency, we co-cluster users based on hashtag frequency within their tweets. To do so we develop *W*_*V*_*t*_×*V*_*n*__, where *w*_*i*,*j*_ ∈ *W*_*V*_*t*_×*V*_*n*__ represents the number of time vertex *v*_*n*,*j*_ appears in the twitter stream of *v*_*t*,*i*_. To co-cluster *v*_*t*,1_…*vt*,*n* we follow the biparitioning algorithm provided in [48], which results in eigenvector features similar to those we defined in the previous paragraph.

The combination of user account attributes, node level metrics from the larger network *G*, and spectral features explained above provide a rich feature space. Paired with a reasonably sized set of labeled vertices, we can detect an extremist community embedded in social media with supervised classification. If labeling vertices is impractical and node attributes appear informative, vertex clustering methods can be used as in [34]. Although we implement two different binary classifiers in Section 3, specific algorithms selected for either phase of this methodology are the decision of the researcher. The end result of IVCC, an accurate extraction of vertices *A*_*t*_, facilitates a social network analysis of the OEC of interest.

## 3 Case study: The ISIS OEC on Twitter

To illustrate the utility of our methodology we offer a case study of the ISIS OEC on Twitter. This case study aims to validate our proposed methodology, present its limitations in terms of ethical use, and provide illustrative examples of intelligence that can be mined from OECs. Although the results of our case study provide strong results in terms of accuracy, and we have provided both traditional and sampling based methods for performance evaluation, we stress that we see these methods primarily as a means to understand the interests and behaviors of this OEC. As with any classification technique, false identification of ISIS OEC members must be considered by the practitioner, and using IVCC to support any type of intervention should be used within the context of multiple sources of intelligence. We discuss intended use and the societal implications of similar methodologies in detail in Section 4.

### 3.1 ISIS data

In this section we describe both our collection methods and dataset, but before doing so we would like to clearly state that we have complied with all of Twitter’s terms of service and privacy policies [49]. We also make no attempts to bind online and offline identity, and have de-identified all users in the data shared in within this manuscript. As a result no ethics or IRB approval was obtained or required. To develop our dataset, we instantiate our sampling strategy with five known, influential ISIS propagandists highlighted in [50]. In November, 2014 we conducted a two step *snowball sample* [51] of these users’ following ties using the Twitter REST API. Snowball sampling is a non-random sampling technique where a set of individuals is chosen as “seed agents.” The *k* most frequent accounts followed by each seed agent are taken as members of the sample. This technique can be iterated in steps, as we have done in our search. Although this technique is not random and prone to bias, it is often used when trying to sample hidden populations [11].

Step one of our search collected user account data for our 5 seed agents’ 1345 unique following ties. Step 2 resulted in account information for all users followed by the 1345 accounts captured in step 1. Our search resulted in 119,156 user account profiles and roughly 862 million tweets. This network is multimodal, meaning that it has two types of vertices, and multiplex, because it has multiple edge types. We represent this set of networks, as a heterogeneous social network with annotated nodes [52], *G* with two node classes: users and hashtags, and four types of links: following relationships, mention relationships, and user-hashtag links. Summary statistics of each network are provided in Table 1.

**Table 1:** Depicts *G*_*full*_, the resultant heterogeneous network from our 2-step snowball search of known ISIS OEC members. The search yielded 400 G of data containing 119,156 Twitter user accounts’ following ties, account profiles, and tweets.

Metric	Network				
	*F* Following	*F*_*rec*_ Reciprocal Following	*M* Mention	*M*_*rec*_ Reciprocal Mention	*H*_*userxuser*_ User by Hashtag
From Node To Node	User User	User User	User User	User User	User Hash Tag
Link Type	directed, binary	undirected, binary	directed, weighted	undirected, weighted	undirected, weighted
Nodes	119 k	119 k	109 k	109 k	106 k x 4 M
Links	23.1M	3 M	14.6 M	1.1 M	27.4 M
Density	0.00163	0.000425	0.00123	0.00018	0.000065
Isolates	0	10888	291	30,047	0
Dyads	0	104	6	425	188
Triads	0	19	0	50	33
Larger	1	8	2	7	6

The snowball method of sampling presents unique and important challenges within social media. Users’ social ties often represent their membership in many communities simultaneously [53]. At each step of our sample, this results in a large number of accounts that have little or no affiliation with ISIS. The core problem of the present work is to identify the set of users within the 119,156 accounts collected that support ISIS in varying degrees. In order to do so, we required a rigid definition of what it means to support ISIS. We define the following three user types of interest:

- *ISIS OEC member*: Similar to [11], we code users who unambiguously support ISIS as OEC members. For example, if the user positively affirmed ISIS leadership or ideology, glorified its fighters as martyrs, affirmed ISIS’ call to Jihad as a duty for all Muslims, or used pro-ISIS images in their profile (i.e. the ISIS flag or images of key figures like Abu Musab Al-Zarqawi or Abu Bakr al Baghdadi), we coded them as OEC members. Furthermore, in light of the growing emphasis placed on “passive observers” [9], we infer retweets as endorsement. Therefore, a member’s *support* is relative and in many cases not in violation of local law or Twitter’s terms of use. However, including this broad continuum of support facilitates the study of populations that could be more susceptible to radicalization.
- *non-member*: A user whose tweets were either clearly against ISIS or showed no Jihadist content.
- *official account*: We label vertices as *official accounts* if they meet any of the following criteria: the user’s account identifies itself as a news correspondent for a validated news source; the account is attributed to a politician, government, or medium sized company or larger; or, following [11], if the account has more than 50,000 followers. This third categorization was deemed necessary as in the process of our case study, we identified dense following and mention ties between ISIS OEC members and news media, politicians, celebrities, and other official accounts. Such accounts are interesting in that there higher follower counts and mention rates tend to make them appear highly central even though they do not exhibit any ISIS supporting behaviors. *Official Accounts* must be identified and removed for accurate classification of ISIS-supporting, thus illustrating the utility of an iterative methodology.

### 3.2 IVCC implementation

**Fig 2:**
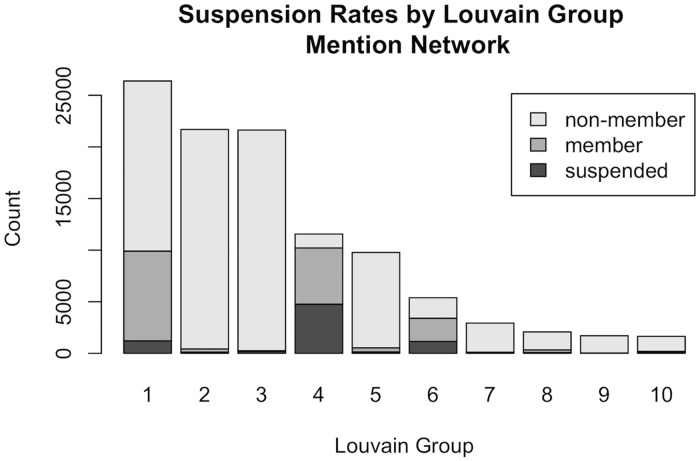
Depicts the size, suspension/deletion rates, and number of users classified as ISIS OEC members within the 10 largest Louvain groups [38] in our weighted, directed network *M* where edges are defined as the number of times user *a* mentions user *b* in his/her Twitter timeline. Our 5 seed agents were assigned to clusters 4 and 6 which had Twitter suspension rates of 41% and 21% respectively. No other cluster had a suspension rate above 5%. Accounts were either deleted by users or suspended by Twitter between the dates of 24 November, 2014 and 12 April, 2015, which coincided with Twitter’s aggressive ISIS related account suspension campaign ongoing in the same time period [54]. We used this combination of factors to select suspended/deleted accounts in groups four and six as training examples of *ISIS OEC members* for classification. It is worth noting that our classifier did not simply find accounts contained in clusters 4 and 6 as is highlighted by the figure as well.

By sampling user accounts from *G* it is clear that the preponderance of accounts collected have no visible affiliation with ISIS, but we, like [11], expect an ISIS supporting community to be captured by our sampling strategy. However, community optimization results of the mention, *M*, and following, *F*, networks highlight an interesting phenomenon. We used the Louvain Grouping method presented in Blondel et al. [38] to cluster *M* and *F*. In each case we found that our 5 seed agents were assigned to one of two clusters. For example, clusters 4 and 6 of the mention network contained all 5 of our seed agents. During the time period between our data collection and analysis, November of 2014 to March of 2015, Twitter has initiated an aggressive campaign to suspend ISIS supporting users [54], and we found the clusters containing our seed agents to have excessively high suspension rates. For example clusters 4 and 6 of the *M* network had suspension rates of 41% and 21% respectively as shown in Fig 2. No other cluster had suspension rates above 5%. Fig 2 depicts the size, suspension/deletion rates, and number of users classified as ISIS OEC members within the 10 largest Louvain groups [38] in our weighted, directed network *M*. We determined excessively high suspension rates within clusters 4 and 6 to be consistent with ISIS support. Although these clusters contained ISIS OEC members, modularity based clustering algorithms like Blondel et al. [38], did not provide enough information to distinguish between ISIS OEC members and other user types. There were still many official and non-ISIS supporting accounts in each of the clusters with elevated suspension/deletion levels, and manual sampling indicated that ISIS OEC members existed in clusters without high suspension rates as well. However, community optimization provided enough context for us to reasonable use the union of suspended/deleted users in Louvain clusters 4 and 6 in *M*, as labelled *ISIS OEC member* cases for vertex classification. Community optimization also helped us identify the need to systematically remove *official accounts*.

We constructed a feature set using spectral representations of the *F*_*rec*_, *M*_*rec*_, and *H*_*user*×*user*;*sharedHashTag*_ networks as described in Section 2. A full list and description of our feature set is included in Table 2. As will be highlighted in Section 4, the ISIS OEC is highly interested in the ongoing operations in Northern Iraq and Syria. As such, they discuss political figures and news sources extensively. Initial attempts to detect the ISIS OEC contained many *official accounts* as previously defined. Therefore, in our first iteration of multiplex vertex classification (MVC) the task was to remove all official, celebrity, and news media accounts. To do so, we conduct an iteration of IVCC by developing a training set of positive and negative examples of *official accounts* to apply to the rest of our dataset. Our positive case labels for official accounts consisted of 2,144 known celebrities, politicians, and journalists as well as an additional 873 accounts with more than 150,000 followers. We labelled the 8,356 suspended/deleted accounts in our dataset as non-official accounts, and trained a *Random Forest* classifier [55] The Random Forest classifier is an ensemble method that constructs a multitude of decision trees and uses the mode of these classes to correct for the problem of overfitting associated with many tree based classifiers. We found its performance to be significantly better than SVM with respect to accuracy when identifying *official* accounts to remove from our dataset. The classifier’s superior performance was likely due to the various types of *official* accounts creating contingencies better captured by a tree based classifier. It is worth mentioning that we are not interested in using this classifier on accounts not contained in *G*; so we conduct use a train/test split, but also use random sampling to assess accuracy.

**Table 2:** Lists and describes features used in each classifier.

Feature:	Source, Description	*θ*_*MNVC*_	*θ*_*SocioDim*_	*θ*_*PMM*_
Creation Date	Twitter User Profile	✓	✓	
Tweet Count	Twitter User Profile	✓	✓	
Follower Count	Twitter User Profile	✓	✓	
Following Count	Twitter User Profile	✓	✓	
Unique Hashtags	Twitter User Profile	✓		
In-Degree Centrality	Follower x Follower Network	✓		
Out-Degree Centrality	Follower x Follower Network	✓		
In-Degree Centrality	Mention x Mention Network	✓		
Out-Degree Centrality	Mention x Mention Network	✓		
Total-Degree Centrality	Follower x Follower Network, Reciprocal Ties	✓		
Total-Degree Centrality	Mention x Mention Network, Reciprocal Ties	✓		
Search Step	Twitter API Script	✓		
*U*_*RF*_	a user x 2 matrix with columns consisting of the eigenvectors associated with the 2 largest eigen values extracted from the graph Laplacian of our Following x Following Network with Reciprocal Ties.	✓		✓
*U*_*RM*_	a user x 2 matrix with columns consisting of the eigen vectors associated with the 2 largest eigen values extracted from the graph Laplacian of our Mention x Mention Network with Reciprocal Ties.	✓	✓	✓
*U*_*UxHT*_	a user x 2 matrix with columns consisting of the eigen vectors associated with the 2 largest eigen values extracted from the graph Laplacian of our User x User (Shared Hash Tag) Network.	✓		✓

The resultant classifier yielded accuracy of 91.3% and an F1 score of 75.8% on these heuristically labeled examples. Our post prediction sampling yielded no significant difference with blind classification of 50 randomly selected accounts. The classifier identified an additional 7,140 news/celebrity/official accounts which we removed from *G* to form *G*^(−)^.

Once we were confident that a high percentage of *official accounts* were removed, we conduct an iteration of MVC to identify ISIS OEC members. For this task we train a Support Vector Machine classifier similar to those presented in [41]. Again, we labeled the 5,126 accounts marked as suspended/deleted and grouped in Louvain clusters 4 and 6 of the *M* network *ISIS OEC members*. We then randomly sampled 10,000 active accounts in Louvain groups 3,4, and 7 in the *F* network and labelled them as *non-ISIS supporting*. The resultant classifier identified 18,335 *ISIS OEC members*. We then combine the classified 18,335 vertices with our 5,126 labelled vertices and construct *A*_*t*_. With our network of suspected ISIS OEC members,*A*_*t*_, we conduct community optimization and network analysis in Section 4. Summary statistics of *A*_*t*_ are provided in Table 3. We acknowledge that our positive case training instances contain uncertainty, as Twitter suspends accounts for a variety of reasons. We will address this issue and discuss our efforts to validate model output in detail if the following section.

**Table 3:** Depicts *A*_*t*_, the suspected ISIS OEC member network identified in Section 4. Each network is more dense than its parent network in *G*.

Metric	Network			
	*A*_*F*_	*A*_*F*,*rec*_	*A*_*M*_	*A*_*M*,*rec*_
Description	Following	Reciprocal Following	Mention	Reciprocal Mention
From Node	User	User	User	User
To Node	User	User	User	User
Link Type	directed,	undirected,	directed,	undirected,
	binary	binary	weighted	weighted
Nodes	21,343	21,343	23,031	22,456
Links	1,254,529	94,583	1.6M	220,597
Density	.0052	.0008	.003	.0004
Isolates	15	1687	269	0
Dyads	2	58	26	0
Triads	0	10	1	0
Larger	1	6	1	0
Reciprocity	.082	1	.016	1
Char. Path Length	3.432	4.44	4.723	15.76
Clustering Coeff.	.129	.154	.111	.065
Network Diameter	11	13	1521	2213

## 4 Performance and validation

In this section we will present our results, first for the model’s performance on our training data set and then we will discuss additional manual validation efforts using our predictions.

Multiplex vertex classification (MVC) extends current methods by applying a combination of the findings developed in [43] and [41]. Given a large multiplex network with annotated vertices, we are able to accurately identify our targeted community, ISIS OEC members. We compare MVC to [43] and [41] by constructing three feature sets:

- *θ*_*MNVC*_: represents the present work and consists of user account features and spectral and node metric representations of the following, mention, and user by user (shared hashtag) networks.
- *θ*_*SocioDim*_: represents [41] and consists of user account features and a spectral representation of the mention network.
- *θ*_*PMM*_: represents Principal Modularity Maximization (PMM) as presented in [43]. PMM utilizes eigenspace representations of the following, mention, and user by user (shared hashtag) networks. For this feature set we used the largest two eigenvectors of each of the respective networks and subsequently performed canonical correlations to maximize the correlations between each network’s respective eigenspaces.

A detailed description of each feature set is provided in Table 2.

Table 4 illustrates *MNVC’s* superior performance across all performance metrics. Accuracy is simply defined as the proportion of correctly classified cases in our test set. Precision is the percentage of positively classified cases that were actually positive. Recall measures the percentage of positive cases that were classified positive. Finally, the F1 Score [56] estimates accuracy by adjusting for bias associated with skewed class distribution. It is important for us to reiterate that our measures of performance in this section quantify how well our classifier was able to differentiate classes in our training data. We acknowledge that we have made assumptions to develop our positive case training instances that could reduce precision when applied to unlabeled data. Therefore, an F1 score of 96% does not necessarily imply that approximately 96% of the users we predict are “true” ISIS-supporting OEC members. However, we have taken measures to validate model output manually as will be explained at the end of this section.

**Table 4:** Performance estimates for the ISIS classifier for feature sets: *θ*_*MNVC*_, *θ*_*SocioDim*_, and *θ*_*PMM*_. The left column depicts both the point estimates and 95% confidence intervals for accuracy. The right column depicts the F1 score [56] associated with each feature set.

Model	Performance Metric	
	Accuracy, 95% CI: Accuracy	F1
*θ*_*MNVC*_	0.96, (0.95, 0.96)	0.93
*θ*_*SocioDim*_	0.87, (0.86, 0.88)	0.80
*θ*_*PMM*_	0.84, (0.83, 0.84)	0.74

We see that *MNVC* outperforms both *SocioDim* and *PMM* with respect to each metric. Although our classifier’s performance is relatively high, with approximately 22,000 accounts classified as ISIS OEC members we would expect more than 900 accounts to be falsely labeled as ISIS OEC members. We will discuss the application of these methods in detail in Section 6. However, a 4% false positive rate and the varying degrees of “support” observed among passive sympathizers again imply these methods would best serve as a means to study online populations that appear vulnerable to online extremism.

With respect to our *official account* classifier, *MNVC* and *SocioDim* performed almost identically. We hypothesize that this is likely due to the heterogeneous nature of *official* accounts. We used this classifier to remove accounts belonging to celebrities, news media, corporations, NGOs, and governmental organizations. Thus, the positive class likely had many contingencies associated with it and would be more well suited to a tree based classifier like the Random Forest algorithm explained in Section 2.

Our use of Twitter suspension rates within specific user groups as positive case labels introduces uncertainty as there are many reasons for Twitter to suspend accounts. To address these limitations, we took several steps to assess the accuracy of our heuristics. This included discussions with native language speakers and blind sampling of accounts predicted as ISIS OEC members. Further, our analysis indicated the ISIS classifier generalizes to unlabeled data in ways that would not suggest biases from our network-based and suspension/deletion-based heuristics. Many of the accounts labeled by our classifier post content that is barbaric and in clear violation of The Twitter Rules precluding the use of the service to promote violence [13]. There are other predicted ISIS OEC members whose content does not clearly violate Twitter’s policies and would generally be considered free speech. However, these users’ content is still consistent with the description of “passive supporters” presented in [9, 10, 12]. Finally, in light of Twitter’s continued aggressive program to remove extremist content from its site [57], we performed an additional check of suspension rates in January, 2017. We found suspension rates of 39%, 7%, and .4% for our predicted classes of ISIS-supporting, non-ISIS-supporting, and official accounts respectively. Although these suspension rates do not conclusively prove any account predicted as an OEC member to be an ISIS-supporter, they do imply that our methodology identifies communities containing sizable pockets of extremism.

We have, in this section, therefore performed a variety of checks to ensure that our classifier is able to identify members of the ISIS OEC in ways that outperform other relevant approaches. As we have noted, there is no way good to assess “ground truth” with pure certainty in our setting, thus leading to some uncertainty in our validation efforts. However, this uncertainty should be considered in the context of many other related tasks in social media mining and natural language processing where the quality of annotation has recently been questioned [58, 59], even on tasks as seemingly straightforward as dependency parsing [60]. While analyses of performance are imperfect here, we have tried in various ways to address them (e.g. through analyzing suspension rates and qualitative analysis of results), making our efforts as stringent if not more so than much related work. Future efforts are needed across the field as a whole in order to better understand how to address these outstanding issues.

## 5 Case study: The ISIS-supporting OEC

The challenge of drawing useful intelligence analyses from social media remains an open research problem, but OEC detection offers new opportunities for intelligence and strategic communications experts to gain needed understanding into large populations susceptible to extremism. The following subsection is intended to provide illustrative intelligence analyses offered by OEC detection.

**Fig 3:**
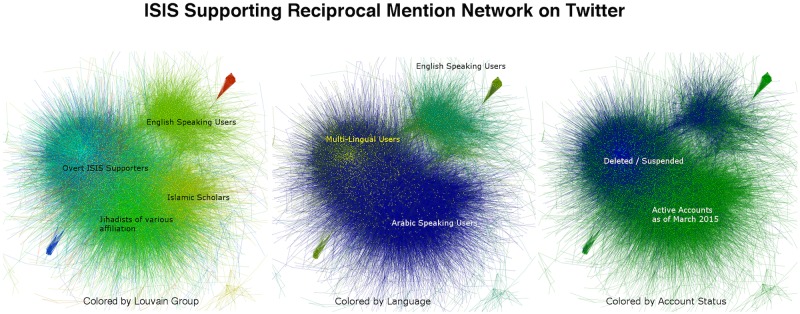
Each panel depicts the ISIS supporting reciprocal mention network, *A*_*M*,*rec*_. The left panel is colored by Louvain Group, the center panel by user language patterns as detected by his or her tweets, and the right panel depicts each user accounts status as of March 22, 2015.

The left panel in Fig 3 depicts the ISIS supporting reciprocal mention network, *A*_*M*,*rec*_, where color indicates Louvain Grouping. Language drives the most clear division among inter-network communities and is highlighted in the middle panel. We used *LangID* as introduced in [61] to identify language at the user level. Blue vertices indicate users whose tweet streams identified as Arabic with probability in excess of 90%, while green vertices depict users whose tweet streams identified as English with probability in excess of 90%. Yellow vertices indicate users whose tweets contain a mixture of English and Arabic. A small portion of those users contained mixed language patterns to include Turkish and Russian. For the most part however, these users form a bridge between the Arabic speaking and non-Arabic speaking communities in the ISIS supporting network.

Interesting structure also exists within the Arabic speaking portion of the community. The relatively small cluster to the far right of the Arabic speaking portion of the community, represented by yellow vertices in the left panel, consisted of accounts sharing lectures and videos on Muslim theology. While the majority of these accounts did not overtly promote jihad or support ISIS, it is interesting to highlight that their follower counts often contained hundreds or thousands of ISIS OEC members. An example of one such account belongs to Dr. Hani al-Sibai who has been cited by Ansar al-Sharia as one of five influential thinkers from whom the terrorists in Tunisia obtain their encouragement [62]. At this time we are unable to determine to what degree these accounts provide active support, or if their followers simply present a fertile recruiting landscape for ISIS propagandists.

**Fig 4:**
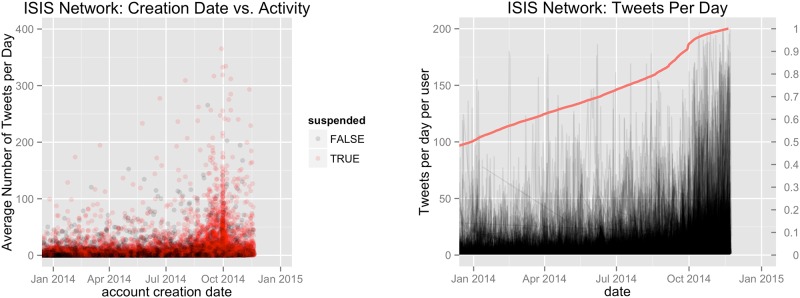
Highlights changes in user activity with respect to time. The left panel depicts ISIS supporting users where the *x-axis* depicts account creation date, and the *y-axis* depicts the average number of tweets per day for the life of the account. Color indicates the suspension status of the account where a black circle indicates the account remains active, while red indicates the account has been deleted or suspended. The right panel depicts 10,000 randomly sampled, ISIS supporting tweet streams in black. Each time series has a high level of transparency to illustrate the distribution of daily user activity over time. The red line depicts the cumulative distribution function of account creation dates within the ISIS supporting network.

It also appears that some propagandist accounts use bots to gain stronger influence. The red and blue groups depicted in the left panel of Fig 4 are visible examples of what we believe to be bots in our dataset. We believe these to be bots because in each case the groups represent a fully connected sub-group where each account repeatedly mentions all other members of the group, as well as a ‘parent account’ or accounts. Although relatively few accounts exhibit this group structure, we hypothesize they are used to elevate the relative popularity of the associated “parent accounts” and remove them for subsequent analysis.

Fig 4 highlights changes in user activity with respect to time. The left panel depicts ISIS supporting users where the *x-axis* details the account creation date and the *y-axis* gives the average number of tweets per day for the life of the account. Color indicates the suspension status of the account, where a black circle indicates the account remains active, while red indicates the account has been deleted or suspended. The right panel depicts a time series of the tweet stream of 10,000 randomly sampled ISIS supporting users (black lines). Each time series has a high level of transparency to illustrate the distribution of daily user activity over time. The red line depicts the cumulative distribution function of account creation dates within the ISIS supporting network. The plot highlights the creation of many ISIS supporting accounts providing a high volume of tweets in the fall of 2014. In particular, the large number of high tweet volume accounts introduced in early October 2014 were likely bots. Though the left panel clearly highlights Twitter’s ability to identify and suspend these accounts, their effect is clearly seen in the right panel, and this highlights the group’s use of bots to possibly generate recruits and/or inflate the perception of their appeal.

Beyond understandings of the group structure and tweet time series, the role and relative importance of users within the observed social network network are of interest. To gain insight into this, we rely on two link types within our dataset: mention and following ties. Reciprocity has been shown to be a strong indicator of trust within online social networks [44–46], and reciprocal mention ties provided the most information gain with respect to our ISIS supporting classifier. Co-mention ties also provide strong indicators of core membership within our ISIS supporting network. Both betweenness and degree centrality quantify how “trusted” a user is among other members of the network, but trust alone does not identify core members or help distinguish roles. To account for this we construct the following metric, which quantifies the proportion of a user’s following ties that are members of our ISIS supporting network, *A*. We refer to this metric as *ISIS Focus*, and use it as a proxy for the user’s ideological affiliation with ISIS.

$$\begin{array}{c} \text{ISIS}\;\text{Focus}=\frac{f_{\text{ISIS}\;\text{Supporting}}}{f_{total}} \end{array}$$ (2)

**Fig 5:**
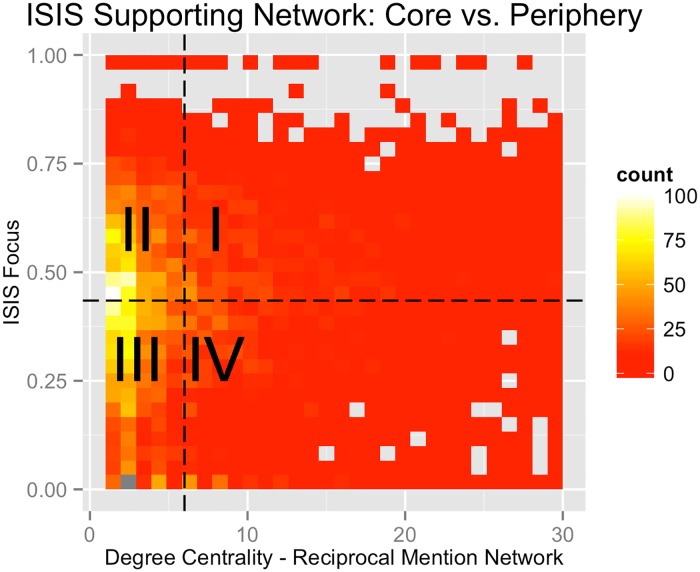
Depicts the distribution of ISIS supporting accounts with respect to degree centrality in the reciprocal mention network (*x-axis*) and ISIS focus (*y-axis*). ISIS focus refers to the proportion of an individual user’s following ties that are classified as ISIS supporting. The dashed white lines depict the median values their respective metrics.

Fig 5 depicts the bivariate distribution of users classified as *ISIS supporting* with respect to degree centrality within the reciprocal mention network (*x-axis*) and ISIS focus (*y-axis*). The dashed black lines depict the median values of the two respective metrics, dividing the plot into four quadrants. Though the quadrants depicted in Fig 5 do not represent finite delineations with respect to user role type, we find that both metrics provide useful information when identifying core members.

Users with high degree centrality and high ISIS focus (quadrant I in Fig 5) are powerful disseminators of ISIS’ message. These are often accounts of popular fighters, accounts designed to look like legitimate news media, or simply popular ISIS propagandists. Those with high ISIS focus and low degree centrality (quadrant II) represent similar accounts, but with less popularity. They appear to have ideals almost identical to those in quadrant I, but are either less skilled at generating a following or relatively new to the network. We also expect recruits to be more likely identified in quadrant II. Accounts with high degree centrality and low ISIS focus are highly trusted but not as highly affiliated with ISIS. This quadrant contained accounts that did not overtly support ISIS but provide information highly relevant to core members like regional news media and Islamic sermons and educational material. Additionally, there were users who appeared loyal to other jihadist groups such as Jabhat al Nusra or Ahrar al Sham or other popular causes in the region such as charities associated with Gaza. Finally, users with relatively low scores in both metrics (quadrant III) represent passive observers.

These measures are important in that we can use these measures to prioritize additional searches of the Twitter API. That is, for those users we identified in Step 2 of our sample, we have not collected accounts from all of their following ties, and can now use a combination of these metrics to prioritize which accounts to download.

**Fig 6:**
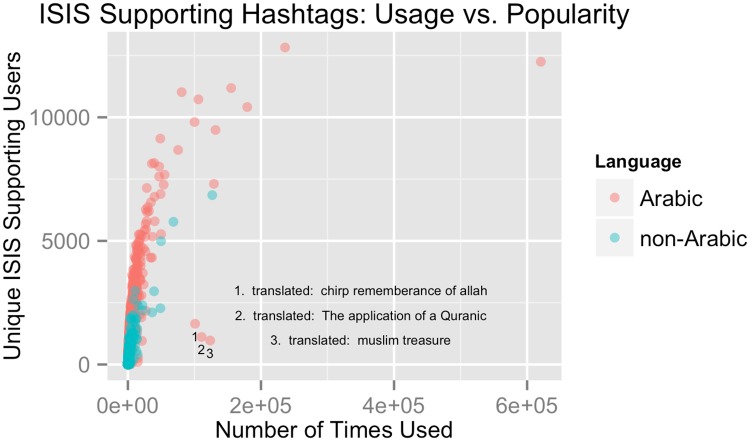
Depicts the 369,603 unique hashtags used by ISIS OEC members in our dataset. Black points depict Arabic hash tags, and red points depict hashtags in other languages. Generally, a hashtags frequency and the number of unique users tagging with it are proportional; however some hashtags, like the three labelled in the figure, seem to have frequencies inflated by a relatively small, highly active group of users.

Removing non-ISIS supporting accounts also enables us to understand the topical interests of ISIS OEC members and how they evolve over time. Such analysis is critical to gain understanding and counter ISIS’ narrative and its ability to generates resources. We quantify both the frequency of various hashtags, as well as the number of distinct actors using them. This enables us to identify what topics have the broadest appeal, as well as topics that might be the result of a small set of highly active users. Fig 6 depicts the 369,603 unique hashtags used by ISIS OEC members in our dataset. Blue points depict Arabic hash tags, and red points depict hashtags in other languages. Generally, a hashtags frequency and the number of unique users tagging with it are proportional; however some hashtags, like the three labelled in the figure, seem to have frequencies inflated by a relatively small, highly active group of users. A closer look at hashtag 1 is translated “Tweet mentions of Allah” and is associated with a Twitter application that offers to mention God every hour on a user’s timeline. The hashtag is used over 100,000 times but by a relatively small set of 1648 users. Of these tweets, 75,382 are posted by only 100 users who all seem to retweet one another’s verses from the Quran and Hadith as well as unique ISIS related content from the battlefield. In other words these hashtags are used by high volume tweeting users to systematically link the groups theology with battlefield exploits. We postulate that this type of analysis could also identify key mouthpieces or propagandists in the network.

**Fig 7:**
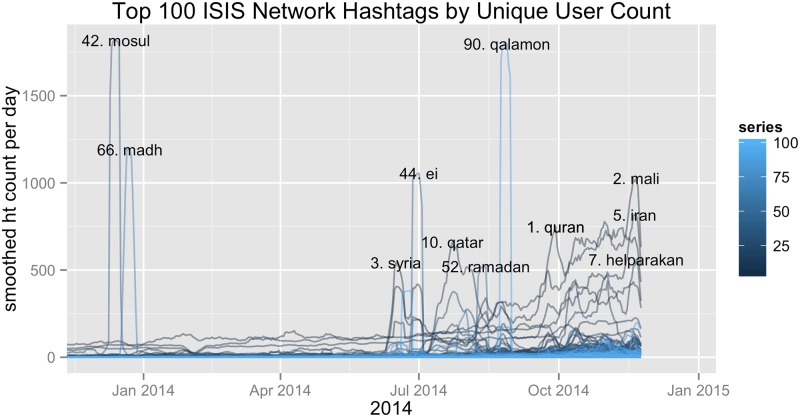
Depicts the smoothed time series of the top 100 ASCII character hashtags in terms of number of unique users. The series are calculated using a 7 day moving average of each respective hashtags frequency in our ISIS supporting network. All hashtags whose average is greater than 500 at any given time are labelled.

More broadly, we can identify the most unifying and energizing topics of the network by looking at how the most broadly used hashtags change over time. Fig 7 depicts the top 100 non-Arabic hashtags in terms of number of unique ISIS supporting users. The y-axis depicts the seven day moving average of the respective hashtags frequency over time. Non-Arabic hashtags with a moving average that reach above 500 tweets per day at any given time period are labelled.

Many of the popular hashtags confirm things we already know about the ISIS supporting movement. ISIS OEC members focus on events relating to Sunni conflict in the greater MENA region, and the temporal peaks in Fig 7 reflect those interests. However, some of these hashtags offer novel insight. For example, the popularity of *#helparakan*, referring to a state in Burma, is consistent with the ISIS Study Group’s assertion that expansion into South Eastern Asia is one of ISIS’ strategic objectives [63]. The trending hashtag *#EI* refers to ‘l’etats Islamic’ and highlights the networks interest in Mehdi Nenmouche, a French jihadist’s arrest and pending extradition to Belgium in June of 2014 [64]. Identifying these topics of interest and the influential users tweeting about them could provide useful understanding of the group’s ‘marketing’ objectives and help drive intervention strategies.

## 6 Societal implications and methodological limitations

The responsible use of social media intelligence and its relationship to individual privacy in democratic states is an important, open question for policy makers [19, 65, 66]. To this end, we acknowledge that our methods could be unethically employed to identify political opposition or dissidents. Indeed, our classifiers that did not incorporate analysis of hashtags routinely identified online activism related to a variety of causes.

Consequently, we join Walsh et al. in their advocation of patient, nuanced political dialog with respect to developing open source intelligence policy in Western democracies [19]. This policy debate centers around both social media users’ reasonable expectation of privacy and the ethical implications of mining their online content.

With respect to the latter, the ethical implications of mining online content using our method vary based on the intended use of the method. We have envisioned here two use cases for IVCC. First, and most importantly, as Western governments have started to search for diplomatic means to counter extremist propaganda, IVCC can be as a means to gain understanding of online populations vulnerable to extremism. We believe this to be an ethical use of the method, as the primary intention is to reduce the likelihood of an individual being deceptively coerced into an extreme ideology. A second use case of IVCC would be for intelligence collection. This use case certainly could require more restrictive policy depending to intelligence category.

With respect to the former element of policy debate, it is without question that users’ reasonable expectation of privacy must be kept in mind at all times. A common argument against doing so is that social media users have the ability to privatize their accounts, or to not use the media at all. However, these options are often not tenable. Further, although many users understand their online behavior is used for marketing purposes, they may not be comfortable with their behavior being used to inform diplomacy or military operations. Indeed, one could assume users would not consent to the use of their information for intelligence collection.

This distinction between marketing versus intelligence objectives in an important one, particularly in light of the mission statement for the newly formed United States Department of State’s Center for Global Engagement:

*The State Department is revamping its counter-violent-extremist communications efforts through a new Global Engagement Center. This center will more effectively coordinate, integrate and synchronize messaging to foreign audiences that undermines the disinformation espoused by violent extremist groups, including ISIL and al-Qaeda, and that offers positive alternatives. The center will focus more on empowering and enabling partners, governmental and non-governmental, who are able to speak out against these groups and provide an alternative to ISILs nihilistic vision. To that end, the center will offer services ranging from planning thematic social media campaigns to providing factual information that counters-disinformation to building capacity for third parties to effectively utilize social media to research and evaluation*. [67]

For objectives similar to those listed above, the use of IVCC by government agencies would therefore be subject to similar protocols to those used for behavioral research by institutional review boards (IRBs). These protocols include a strong push for de-identification—our methods make no attempt to bind online and offline identities, and agencies using these methods to inform messaging efforts could do so with de-identified data. While we acknowledge that the use of bulk de-identified meta-data has been the subject of concern [19], this issue is routinely encountered by IRBs in academia as well.

Further, within a context of informed diplomatic messaging, the use of IVCC is thus proximal to academic research and further, analogous to individually tailored online marketing. Ethical employment of our methods could be carried out to understand vulnerable online populations and ultimately preserve civil liberties. Peacetime military information operations aimed at messaging to specific populations could be viewed similarly [68], and implemented with de-identified data.

The complexity of these issues requires a substantive theoretical framework under which to characterize these various ethnical concerns. Walsh et al. (2016), who provide a framework with which to balance the importance of civil liberties with national security in an intelligence context [19]. Their framework is based on the collection method, context, and target. In our case, social media mining would be our method; however the increasingly complex combinations of context and target imply the need for nuanced policy. Currently, policy has started to address the varying expectations of privacy in wartime, peacetime, and counter-terrorism contexts. However, the onset hybrid warfare that is conducted by state and non-state actors purposely beneath the threshold of Western military intervention [69] further complicates policy development.

The intelligence target also has policy implications. Specifically, the purpose and category of the desired intelligence product needs to be considered. For the purpose of describing a commander’s operating environment or assessing ongoing operations, authorities could be quite liberal. Intelligence used to develop military targets or bind online and offline identity would imply more restrictive policy. As stated by Walsh et al, the increasingly complex nature of conflict call for patient political dialogue, and policy makers need to ‘take their citizens with them’ when making arguments for new policy and authorities [19].

In sum, implementation of IVCC for social media intelligence does, on the one hand, require a more formal framework and more nuanced discussion. On the other hand, however, it is clear that the method can also be used in many ethical fashions and to improve efforts of national security.

## 7 Conclusion

The present work makes two major contributions to the literature. First, we develop iterative vertex clustering and classification (IVCC), a scalable, annotated network analytic approach for extremist community detection in social media. Our approach outperforms two existing approaches on a classification task of identifying ISIS supporting users by a significant margin. Second, we provided an illustrative case study of the ISIS supporting network on Twitter. To the best of our knowledge, it is the most comprehensive study of this network, and it provides a variety of important insights that may prove important in better understanding the incredible proliferation of ISIS propaganda on Twitter. Most notably, we find that:

- Leveraging the multiplex and multinode structures available in Twitter data significantly improved our algorithm’s ability to accurately identify ISIS OEC members on Twitter.
- Identifying and isolating large portions of an online extremist community offers unique insights into the group’s ideology and influence, and helps identify key users and roles.
- IVCC offers promise for making online extremist community detection in social media a practical reality to inform both diplomacy and defense initiatives.

This case study offers a unique opportunity to infer positively labelled cases based on Twitter suspensions and clustering techniques. However, it is unlikely that such a large number of labeled cases would always be available. Thus, implementations using semi-supervised algorithms or active learning [70] would make IVCC more generalizable, and should be a topic for future research. IVCC is also limited in that it does not account for simultaneous group membership of users. It is likely that there are jihadists that support various terrorist groups and allegiances can be dynamic. IVCC does not provide probabilistic clustering or account for changes in group dynamics over time. Similar to [71], we would like to extend this methodology to an overlapping group framework to account for these types of users and also explore methods to identify temporal change points. Finally, though preliminary results for IVCC as a methodology are encouraging, they are limited in that we do not provide an empirical analysis of IVCC with respect to benchmark. We will leave this analysis to future work, due to the emphasis of this paper being the ISIS case study.

Extremist community detection is an important need in processing social media, and with such approaches like IVCC, we hope that the influence of groups like ISIS can be counteracted in the near future.
